# Organizational Factors and Long-Term Mortality after Hip Fracture Surgery. A Cohort Study of 6143 Consecutive Patients Undergoing Hip Fracture Surgery

**DOI:** 10.1371/journal.pone.0099308

**Published:** 2014-06-13

**Authors:** Caterina A. Lund, Ann M. Møller, Jørn Wetterslev, Lars H. Lundstrøm

**Affiliations:** 1 Department of Anaesthesia and Intensive Care, Herlev University Hospital, Herlev, Denmark; 2 Copenhagen Trial Unit, Centre for Clinical Intervention Research, Rigshospitalet, Copenhagen University Hospital, København, Denmark; 3 Department of Anaesthesia, Nordsjællands Hospital, Hillerød University Hospital, Hillerød, Denmark; D'or Institute of Research and Education, Brazil

## Abstract

**Objective:**

In hospital and health care organizational factors may be changed to reduce postoperative mortality. The aim of this study is to evaluate a possible association between mortality and ‘length of hospital stay’, ‘priority of surgery’, ‘time of surgery’, or ‘surgical delay’ in hip fracture surgery.

**Design:**

Observational cohort study.

**Setting:**

Prospectively and consecutively reported data from the Danish Anaesthesia Database were linked to The Danish National Registry of Patients and The Civil Registration System. Records on vital status, admittance, discharges, codes of diagnosis, anaesthetic and surgical procedures were retrieved.

**Participants:**

6143 patients aged more than 65 years undergoing hip fracture surgery.

**Main Outcome Measures:**

All-cause mortality.

**Results:**

The one year mortality was 30% (28–31%, 95% Confidence interval (CI)). In a multivariate model ‘length of hospital stay’ less than 10 days and more than 20 days are associated with mortality with hazard ratios of 1.34 (1.20–1.53 CI, p<0.001) and 1.27 (1.06–1.51 CI, p<0.001), respectively. ‘Priority of surgery’ categorized as ‘non-scheduled’ is associated with mortality with a hazard ratio of 1.31 (1.13–1.50 CI, p<0.001). Surgical delay and time of surgery are not significantly associated with mortality.

**Conclusion:**

Non-scheduled surgery and length of hospital stay were associated with increased mortality. Confounding by indication may bias observational studies evaluating early and late discharge as well as priority; therefore cluster randomized clinical trials comparing different clinical set ups may be warranted evaluating health care organizational factors.

## Introduction

Mortality after hip fracture surgery is high and has been reported to range from 14 to 36% after one year [1]. To predict and reduce mortality after surgery for hip fracture, several patient related risk factors have been identified [1]–[4]. Most of these risk factors cannot be altered, whereas in hospital organizational risk factors may be changed in the individual hospital daily routine to reduce morbidity and mortality. However, the knowledge of the importance of different organizational factors remains inconclusive. The administrative management consist of interventions strongly depending on numerous conditions related to the patient, the physician etc. As an example both ‘surgical delay’ and ‘length of hospital stay’ is probably affected not only by organizational (i.e., the time required for the diagnostic procedures) but also clinical (i.e., the development of intercurrent adverse clinical events) factors. The impact of surgical delay on mortality is uncertain [5]–[16]. Furthermore, length of hospital stay has primarily been evaluated as an outcome measure [2]; [14]; [17]; [18] rather than a determinant for mortality. In this large cohort study with prospectively and consecutively recorded data, we evaluated the impact of factors partly categorized as organizational such as ‘surgical delay’, ‘length of hospital stay’, ‘time of surgery’ and ‘surgical priority’ on long-term mortality after hip fracture surgery.

## Material and Method

The Danish National Board of Health, The Danish Data Protection Agency and The Danish Ethics Committees for Biomedical Research approved the registration of data in the Danish Anaesthesia Database. The Danish Data Protection Agency (Capital Region journal number 2007-58-0015) and the steering committee of the Danish Anaesthesia Database approved this study and provided access to the data for the analysis presented here. Since this is a database study, informed consent from the individual patient is not needed according to Danish legislation. Danish Database studies should not be sent for ethical approval, only be approved by the institutions mentioned above.

### The data sources

#### The Danish Anaesthesia Database

Fourteen Danish departments of anaesthesia in 2005, and 25 departments in 2006–07, prospectively and consecutively reported data to the Danish Anaesthesia Database version 2 concerning all patients undergoing anaesthesia for surgery. The Danish Anaesthesia Database contains specific quantitative anaesthetic and surgical indicators describing the perioperative period. The departments (see Appendix I) are connected via the Internet to a central server hosted by The Unit for Clinical Quality, the Capital Region, Denmark. The information is recorded immediately after each anaesthesia and surgical procedure. The interface of the database is interactive and changes depending on the type of anaesthesia and surgery that is registered. All registered parameters are predefined and the interface to register was the same for all the registration sites as well as the rules of validation and the on-line user manual. Each patient entered into the database is registered with a unique identifying number from the centralized civil register. To construct the inpatient history, data from the different data sources were matched by this unique 10-digit national identification number that is assigned to every Danish citizen at birth. Furthermore, this unique identifier contains information regarding the patient's sex and date of birth and enables registration of each patient during the statistical analysis and prevents duplicates of anaesthesia records.

#### The National Registry of Patients

Contains information on admittance's, discharges, codes of diagnosis and surgical procedures from public hospitals in Denmark.

#### The Civil Registration System

Contains information on the vital status of all Danish citizens.

### Data processing and study population

**Figure 1:**
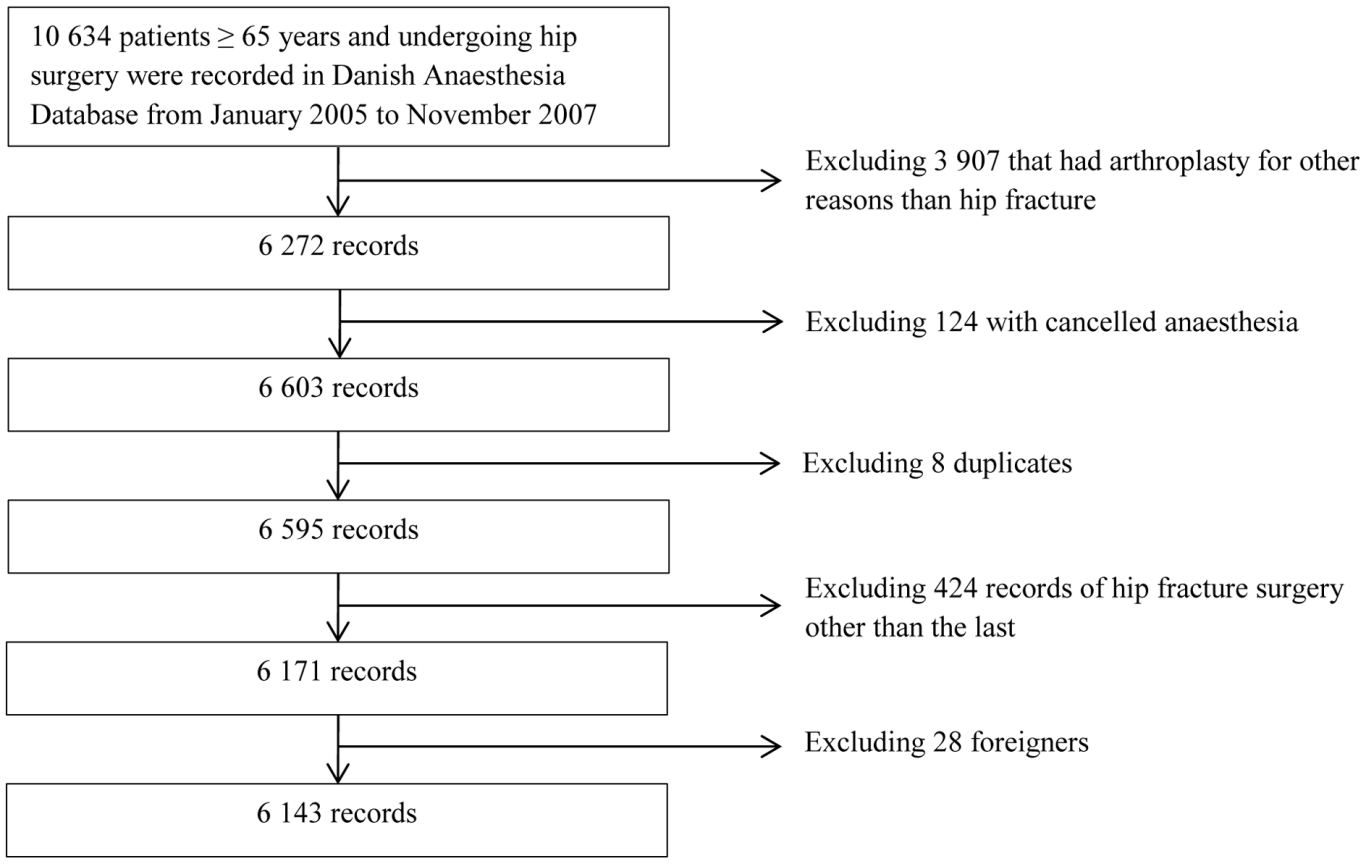
Selection of the study cohort.

The cohort included patients recorded in the Danish Anaesthesia Database from January 2005 to November 2007 who were aged 65 or older and undergoing surgery following hip fracture. In the Danish Anaesthesia Database 10 634 patients were identified according to the “Nordic Classification of Surgical Procedures” records with codes for internal fixation (KNFJ50/1/2, KNFJ60/1/2, KNFJ70/1/2, KNFJ80/1/2) or arthroplasty (KNFB12, KNFB02, KNFB20, KNFB30, KNFB40). By using the patients unique identification number the codes of surgical procedure retrieved from the Danish Anaesthesia Database were linked with their corresponding diagnostic action codes in Nordic Classification of Surgical Procedures. We used codes from a Danish version of World Help Organization's “International Statistical Classification of Diseases and Related Health Problems version 10”: DS720, DS721, DS722 to identify patients that had surgery for intracapsular, peritrochanteric or subtrochanteric fractures, respectively. Hereby we excluded 3907 patients who were treated with an arthroplasty for other reasons than acute fracture. A total of 424 patients were undergoing surgery because of hip fracture on more than one occasion, for these patients only the last record was included. The vital status of all patients was obtained by The Civil Registration System on January 14, 2008. For 28 foreign patients who were recorded in Danish Anaesthesia Database but had left Denmark before the vital status check in The Civil Registration System, the vital status was not available. These patients were excluded from the cohort. The final study population consisted of 6143 patients (figure 1).

### Covariates

Covariates were obtained by the Danish Anaesthesia Database, the National Registry of Patients and the Civil Registration System. The covariates concerning organizational factors undergoing primary assessment were ‘length of hospital stay’, ‘priority of surgery’, ‘time of surgery’, and ‘surgical delay’. Further, the following other covariates were retrieved for our assessments: age; sex; body mass index [19]; American Society of Anesthesiology classification of physical status [20]; day of admission; type of surgery; previous hip fracture surgery; type of fracture; duration of surgery; time of the year; type of hospital, type of anaesthesia and duration of surgery.

‘Length of hospital stay’ (LOS) was defined as the time between admission and discharge from the hospital. From a clinical point of view we anticipated, *a priori*, that a ‘Length of hospital stay’ of 10 days to two weeks would be recognized as usual and that ‘Length of hospital stay’ lesser than that would be anticipated as either fast recovery or if the patient dies as unsuccessful recovery and that ‘Length of hospital stay’ beyond 15 days would be recognized as a lengthy stay. Thus it was stratified as: ‘length of hospital stay’ <10 days, 10 to15 days, 16 to 20 days and 20 days < ‘length of hospital stay’). Priority of surgery was defined as non-scheduled if a patient was anaesthetized without being planned for surgery the previous day. Otherwise surgery was categorized as scheduled. Time of surgery was categorized as ‘dayshift’ if start of surgery was between 08:00 and 16:00 or as ‘nightshift’ if start of surgery was between 16:00 and 08:00. The surgical delay was defined as the length of time between the admission to the department of surgery and the starting time of hip fracture surgery. We stratified surgical delay as: <12 hours; 12 to 23 hours; 24 to 47 hours; 48 to 71 hours; 72 to 95 hours; ≥96 hours. Age was categorized as (65 to 69 years; 70 to 79 years; 80 to 84 years; 85 to 89 years; ≥90 years). Body Mass Index was calculated as weight · height^−2^ (kilogram · meter^−2^) and categorized as: underweight, Body Mass Index <18.5; normal, 18.5≤ Body Mass Index <25; overweight, 25≤ Body Mass Index <30; obesity, 30≤ Body Mass Index. Day of admission was categorized as ‘week-end’ if the patient was hospitalized Friday, Saturday or Sunday, while admission during rest of the week was categorized as ‘not week-end’. Time of year was defined by the date of surgery according to the season. Type of surgery was categorized as either KNFB (arthroplasty) or KNFJ (internal fixation). ‘Previous hip fracture surgery’ was defined as the patients who were undergoing surgery because of hip fracture more than once during the time of observation and was categorized as ‘Yes’ or ‘No’. Based upon international code of disease 10 codes: DS720, DS721, DS722 the type of fracture was categorized as intracapsular, peritrochanteric or subtrochanteric, respectively. Duration of surgery was measured in minutes, and categorized as: <60 minutes; 60 to 90 minutes; >90 minutes. Type of anaesthesia was defined as general, regional or combined general and regional. Hospitals were divided into 3 groups based on the number of operations performed during one year (2007) of observation and stratified as: (<100, 100 to 199, >199).

### Statistical Analysis

The overall mortality rates for two; six and 12 months were reported. The associations between death and the predefined covariates were assessed by Cox proportional hazards regression analysis. The primary analysis used start of the follow up at admission and a sensitivity analysis using start of follow-up after discharge from hospital. Initially, univariate regression analyses were performed. Subsequently, all significant covariates from the univariate analyses were included in a multivariate regression analysis. Backward stepwise regression was performed to identify a final Cox model. Survival functions were displayed for the organizational covariates contained in the final model. The assumption of proportional hazards was checked plotting cumulative hazard functions for different categories of ‘length of hospital stay’, ‘priority of surgery’ and sex. The cumulative hazard functions as well as the log minus log plots were very close to parallel and did not suggest violation of the assumption of proportional hazards. Because the association of ‘length of hospital stay’ with mortality may depend on the in-hospitality mortality, we performed a sensitivity analysis. Here we used the same co-variates as in our primary analyses, but excluded all patients who died during hospital admission. All estimates were reported with 95% confidence intervals.

The prevalence and pattern of missing data among all covariates were examined. If more than 10% of the patients had missing records for one or more covariates, the statistical method of multiple imputations for handling missing data [21]–[23] was used. Otherwise the results of the original dataset of complete cases were presented. For both the univariate and multivariate regression analyses we used a level for statistical significance of p<0.05. The data were analysed using the SPSS version 16.0 (SPSS Inc., Wacker Drive, Chicago, IL 60606-6307).

This study has been presented according to the STROBE-statement of reporting of an observational cohort study [24].

### Ethical approval

The Danish Ethics Committees for Biomedical Research approved the registration of data in the Danish Anaesthesia Database.

### Data sharing

No additional data available.

## Results

From January 2005 to November 2007, 6 143 patients recorded in Danish Anaesthesia Database met the criteria for inclusion in this study. A total of 469 (7.6%) of the 6 143 patients had missing records for one or more covariates, whereas 5 674 patients had complete records without any missing data. The characteristics of the patients are shown in Table 1. The mortality rates after two, six and twelve months were 15.2% (14.3–16.1, 95% CI), 23.4% (22.2–24.6, 95% CI) and 29.8% (28.3–31.3, 95% CI), respectively. Performing Cox proportional hazards regression analysis, the median follow-up time was 9.8 months with a 90% inter percentile range from 0.3 to 30.8 months.

**Table 1:** Characteristics of the 6 143 patients.

Covariates		Number of patients (%)	Covariates		Number of patients (%)
**Sex**			**Priority of Surgery**		
	Male	1597 (26)		Scheduled	1097 (17.9)
	Female	4546 (74)		Non-scheduled	5046 (82.1)
	Missing	0		Missing	0
**Age – years**			**Time of Admission**		
	Age <70	786 (12.8)		Week-end	2367 (38.5)
	70≤ age <80	1538 (25.0)		Not week-end	3710 (60.4)
	80≤ age <85	1381 (22.5)		Missing	66 (1.1)
	85≤ age <90	1349 (22.0)	**Time of Year**		
	90≤ age	1089 (17.7)		Spring	1462 (23.8)
	Missing	0		Summer	1597 (26.0)
**ASA**				Autumn	1705 (27.8)
	ASA 1	428 (7.0)		Winter	1313 (21.4)
	ASA 2	3133 (51.0)		Missing	66 (1.1)
	ASA 3	2282 (37.1)	**Surgical delay - hours**		
	ASA 4–6	190 (3.1)		0–12	1151 (18.7)
	Missing	110 (1.8)		12–24	2115 (34.4)
**Body Mass Index (BMI) – kg/m^2^**				24–48	2026 (33.0)
	BMI <18.5	742 (12.1)		48–72	425 (6.9)
	18.5≤ BMI <25	3643 (59.3)		72–96	163 (2.7)
	25≤ BMI <30	1253 (20.4)		>96	197 (3.2)
	30≤ BMI	312 (5.1)		Missing	66 (1.1)
	Missing	193 (3.1)	**Time of Surgery**		
**Previous hip fracture surgery**				Dayshift	4044 (65.8)
	Yes	406 (6.6)		Nightshift	2099 (34.2)
	No	5737 (93.4)		Missing	0
	Missing	0	**Duration of Surgery – minutes.**		
**Type of fracture**				<60	2517 (42.6)
	Intracapsular	3300 (53.7)		60–90	2060 (35.5)
	Peritrochanteric	2409 (39.2)		>90	1335 (21.7)
	Subtrochanteric	341 (5.6)		Missing	131 (2.1)
	Missing	93 (1.5)	**Type of Hospital – operations/years**		
**Type of Operation**				<100	556 (9.1)
	Internal fixation	3993 (65)		100–199	3133 (51.0)
	Arthroplasty	2148 (35)		>199	2286 (37.2)
	Missing	0		Missing	168 (2.7)
**Type of Anaesthesia**			**Length of hospital stay (LOS) - days**		
	Regional	3317 (54.0)		LOS <10	3810 (62.0)
	General	2389 (38.9)		10≤ LOS <15	1067 (17.4)
	Combined	424 (6.9)		15≤ LOS <20	443 (7.2)
	Missing	13 (0.2)		20≤ LOS	636 (10.4)
				Missing	187 (3.0)

ASA  =  American Society of Anesthesiology classification of physical status.

In our univariate Cox regression analyses ‘length of hospital stay’ and ‘priority of surgery’ were significant associated with mortality, while ‘Time of surgery’ (P = 0.20) and ‘Surgical delay’ (P = 0.08) were not significantly associated with mortality. Other significant covariates were Gender; Type of operation; Previous hip fracture surgery; Type of anaesthesia; Type of hospital; Age; American Society of Anesthesiology classification of physical status; Body Mass Index and Type of fracture (Table 2).

**Table 2:** Cox regression analyses.

Covariates	Univariate			Multivariate		
	Hazard ratio	95% C.I.	P value	Hazard ratio	95% C.I.	P value
**Sex**						
Female	Reference			Reference		
Male	1.47	1.34–1.62	<0.001	1.79	1.62–1.99	<0.001
**Age - years**						
Age <70	Reference			Reference		
70≤ age <80	1.58	1.29–1.95	<0.001	1.47	1.19–1.82	<0.001
80≤ age <85	2.12	1.73–2.60	<0.001	1.95	1.58–2.41	<0.001
85≤ age <90	2.95	2.42–3.60	<0.001	2.78	2.26–3.42	<0.001
90≤ age	4.26	3.49–5.19	<0.001	3.86	3.13–4.75	<0.001
**ASA**						
ASA 1	Reference			Reference		
ASA 2	2.46	1.84–3.28	<0.001	2.18	1.63–2.92	<0.001
ASA 3	4.84	3.63–6.45	<0.001	3.85	2.87–5.15	<0.001
ASA 4–6	9.43	6.76–13.16	<0.001	7.76	5.52–10.91	<0.001
**Body Mass Index (BMI)**						
BMI <18.5	1.43	1.26–1.61	<0.001	1.42	1.25–1.61	<0.001
18.5≤ BMI <25	Reference			Reference		
25≤ BMI <30	0.68	0.60–0.77	<0.001	0.73	0.64–0.84	<0.001
30≤ BMI	0.54	0.42–0.70	<0.001	0.61	0.47–0.79	<0.001
**Previous hip fracture surgery**						
No	Reference			Reference		
Yes	1.42	1.15–1.75	<0.001	1.26	1.01–1.57	0.040
**Type of fracture**						
Intracapsular	Reference			Reference		
Peritrochanteric	1.21	1.11–1.33	<0.001	NS		
Subtrochanteric	1.14	0.93–1.39	0.12	NS		
**Type of operation**						
Internal fixation	Reference			Reference		
Arthroplasty	0.80	0.72–0.88	<0.001	NS		
**Type of anaesthesia**						
Regional	Reference			Reference		
Combined	1.03	0.86–1.24	0.72	NS		
General	1.15	1.05–1.26	0.002	NS		
**Priority of surgery**						
Scheduled	Reference			Reference		
Non-scheduled	1.50	1.31–1.71	<0.001	1.31	1.13–1.50	0.003
**Duration of surgery - minutes**						
<60	Reference			Reference		
60–90	0.87	0.78–0.96	0.006	NS		
>90	0.77	0.68–0.86	<0.001	NS		
**Length of hospital stay (LOS) - days**						
LOS <10	1.40	1.23–1.60	<0.001	1.34	1.20–1.53	<0.001
10≤ LOS <15	Reference			Reference		
15≤ LOS <20	1.08	0.88–1.33	0.45	0.99	0.80–1.22	0.913
20≤ LOS	1.47	1.24–1.74	<0.001	1.27	1.06–1.51	0.008
**Surgical delay - hours**						
0–12	Reference					
12–24	0.89	0.78–1.01	0.08			
24–48	1.03	0.91–1.17	0.64			
48–72	1.02	0.84–1.24	0.82			
72–96	1.10	0.83–1.44	0.52			
>96	1.05	0.81–1.36	0.72			
**Time of Surgery**						
Dayshift	Reference					
Nightshift	1.07	0.98–1.18	0.20			

ASA  =  American Society of Anesthesiology classification of physical status; NS  =  non significant. Both Surgical delay, p = 0.08 and Time of Surgery, p = 0.20 were non significant in the univariate analysis. Other Non significant covariates in the univariate analyses were: Time of year, p = 0.67; Time of Admission, p = 0.25; Type of hospital, p = 0.19.

**Figure 2:**
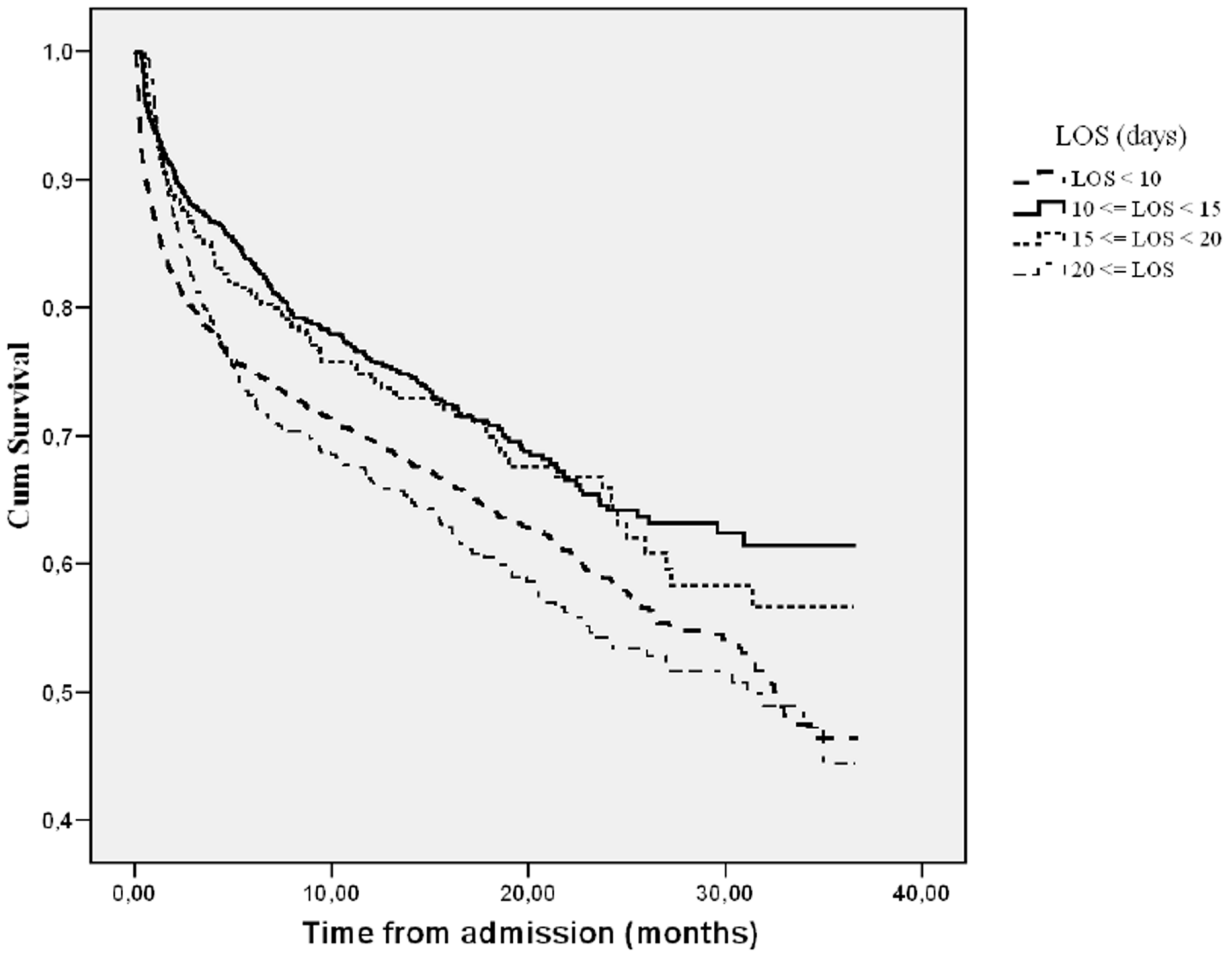
Survival function for ‘Length of Hospital Stay’ (LOS).

**Figure 3:**
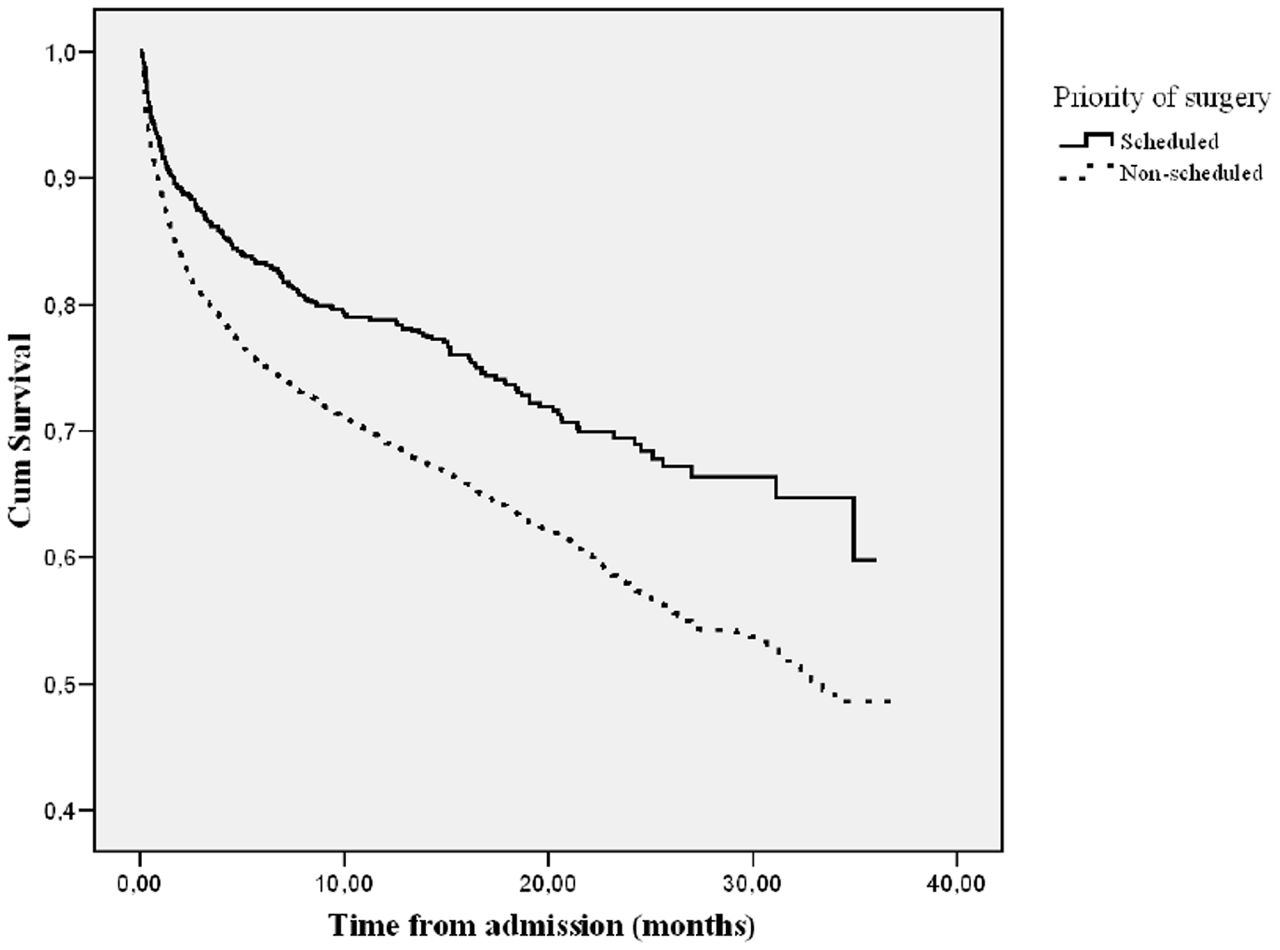
Survival Function for ‘Priority of Surgery’.

In our subsequent multivariate Cox regression analysis ‘length of hospital stay’ and ‘priority of surgery’ remained statistically significant associated with increased mortality. In the multivariate model ‘length of hospital stay’ <10 days and a 20 days ≤ ‘length of hospital stay’ were associated with mortality with a hazard ratio of 1.34 (1.20–1.53, 95% CI, p<0.001) and 1.27 (1.06–1.51, 95% CI, p = 0.008), respectively. The hazard ratio of ‘priority of surgery’ categorized as ‘Non-scheduled’ was 1.31 (1.13–1.50, 95% CI, p = 0.003). The final Cox model includes the following additional covariates: Sex; Previous hip fracture surgery; Type of hospital; Age; American Society of Anesthesiology classification of physical status and Body Mass Index (Table 2). The survival functions of ‘length of hospital stay’ and ‘priority of surgery’ are displayed in figure 2 and 3, respectively.

The impact of ‘length of hospital stay’ on mortality may depend on the in-hospitality mortality (2.0%, (1.6–2.4, 95% CI)). This possible influence was evaluated in an additional sensitivity analysis. With a similar statistical approach as in our primary analyses, we performed Cox regression analyses excluding all patients who died during hospital admission. This additional multivariate analysis did not show any noticeable differences compared with the original analyses, as ‘length of hospital stay’ <10 and a 20≤ ‘length of hospital stay’ were associated with mortality with a hazard ratio of 1.32 (1.25–1.51, 95% CI, p<0.001) and 1.25 (1.05–1.50, 95% CI, p = 0.014), respectively.

## Discussion

We found that neither the surgical delay nor the time of surgery were associated with an increased risk of death. Non-scheduled surgery, early discharge (‘length of hospital stay’ <10 days), and late discharge, (‘length of hospital stay’ >20 days), were statistically significant risk factors for mortality after hip fracture surgery.

The present study is based upon a large cohort of patients with prospectively and consecutively collected data representing everyday experience from clinical practice. The Danish Anaesthesia Database requires all recorded indicators to be subjected to relevant rules of validation. This minimizes subsequent problems of missing and invalid data. The data from *The National Registry of Patients* and *The Civil Registration System* is of a high quality and the unique personal identification number almost ensuring complete follow up of data from Danish Anaesthesia Database concerning date of death after discharge from hospital. The data is recently collected and over a short period of time but with a long follow up time (up to 36.5 months).

There are several limitations in our assessment. Confounding by indication is well known to introduce bias in the results in any non-randomized study involving interventions [25]. In our assessment the ‘organizational covariates’ more and less reflect multiple factors. Especially ‘length of hospital stay’ may depend on various parameters other than organizational factors. Thus, the administrative management consist of interventions strongly depending on numerous conditions related to the patient, the comorbidity, the physician etc. It was not possible to acquire information of any administrative guidelines used by the participating departments these may strongly differ between patients within the same surgical department. As an example, the administrative and clinical choice of discharging a patient could depend on numerous variables not recorded in the Danish Anaesthesia Database, which may hereby bias our results. Even though, the American Society of Anesthesiology classification of physical status [26] was retrieved for our assessment, the mental status, residence and socio-economic status of the patients prior to the injury and after discharge are unknown. Furthermore, the mechanism of injury (pathological fracture, multiples and high energy trauma) and the time between the injury and admission (including inter-hospital transfer) are unknown [27]. All these various factors strongly affect ‘length of hospital stay’ and therefore it is important not to consider this covariate as a “pure” organizational factor. It was a further limitation that it was not possible to retrieve data about the patients' cognitive and functional status before fracture and there was no available information about patient's comorbidity in the database. Finally, we reported all-cause mortality. It would have been of interest to provide the cause of death in the patients during the time of observation, but these information were not contained in the database.

The mortality rates in our study exceed other estimates previously reported [1]; [7]; [8]; [14]; [28]. This might be due to the non-selected patient population including some patients with multiple co-existing diseases. In Denmark, all patients are treated in public hospitals. The present sample is therefore likely to be representative of the Danish population as a whole.

Despite of a trend, we did not find an association between surgical delay and mortality. The knowledge of the importance of surgical delay on mortality remains inconclusive. While some studies have found a decreased mortality with early surgery [6]; [16]; [28]–[31] others have not been able to find an association between surgical delay and mortality after hip fracture surgery [5]; [7]; [9]; [12]; [14]; [18]; [32]–[34]. Comparing the outcomes of these studies is difficult as the cut-offs and definitions of early and late surgery varies between studies. In most studies the number of patients included is small, but even in larger studies the results are ambiguous [7]; [9]; [16]; [28]. Despite of this it is considered good clinical practice in most countries to operate patients with hip fracture within 24 hours after admission. The largest study [6] on this subject included more than 100 000 patients and found an increased risk of death associated with delay. However, this study was limited to in-hospital mortality and therefore it is not comparable with our assessment focusing on long-term mortality. The majority of the patients (87%) in our assessment underwent surgery within 48 hours after admission. Hereby, our patient population differs from most other studies, where the interval of surgical delay was wider [1]; [8]; [12]; [13]; [16]; [28], making them difficult to compare. As an example, patients who in other studies would have been delayed for medical reasons, might have been included in the <48 hours group of our study. It will be difficult to estimate the effect of a possible gain of short delay in interaction with a possible disadvantage of not having optimized the medical status of selected patients.

Short surgical delay may increase the priority of patients with hip fracture on the surgery schedule and influence the time of surgery towards late shift with less experienced surgeons and staff. In our assessment ‘The time of surgery’ was not associated with an increased risk of death. This is in accordance with previous studies on this subject [35]. However, the number of patients undergoing surgery during night shift was limited, and therefore there may not be statistical power to detect a statistical significant association.

We found that patients who underwent non-scheduled surgery had a significant increased risk of mortality. It could indicate that the frailest patients had the highest priority or that the patients were not medically optimized enough for the operation. Thus our results may indicate that surgical precaution including meticulous planning is of importance.

We found that both ‘length of hospital stay’ <10 days and ‘length of hospital stay’ >20 days were independent risk factors for mortality. The increased risk of death with ‘length of hospital stay’ >20 days may be a marker for patients that had deterioration of pre-existing medical diseases or medical or surgical complications during admission. The increased risk of death with ‘length of hospital stay’ <10 days makes us hypothesize that some patients may be discharged too early without the implementation of relevant adjuvant interventions. Fast track systems [36]; [37] focuses on strategies to minimize the length of hospital stay. Short length of stay as an outcome measure might not be at guarantee of quality and cost effectiveness if the patients are not discharged following standardized and optimized discharge criteria. In a multidisciplinary rehabilitation strategy, it may be a goal to reduce the length of hospital stay. However, it is important to understand that discharge criteria with fast-track surgery should be the same as those of traditional care, but that the fast-track system may achieve the criteria sooner [36]; [37]. These interventions including good feeding, physical training, and regular medication may restore physical and mental abilities after general surgery. The increased risk of death with ‘length of hospital stay’ <10 days could suggest that the frailest patients were discharged quickly to nursing homes [38], however the American Society of Anesthesiology classification of physical status was included in the multivariate analysis to correct statistically for their functional status and level of disease. We were not able to retrieve information on clinical criteria for discharge as well as information on out of hospital clinical interventions after discharge. However, a recent national multicenter audit of hospital charts from hospitals in Denmark demonstrated significant variability in treatment and care of patients with hip fractures among the regions of Denmark [39].

In the effort of minimizing the risk of bias caused by confounding parameters, randomized trials are needed. In a randomized trial, it may be methodologically preferable randomizing patients individually [40]. However, in an attempt to assess the impact of the implementation of recommendations made at a departmental level, it may be difficult to conduct organizational interventions at an individual level. Consequently, a cluster-randomized design may be preferable [40]; [41], and hereby different clinical set ups may be compared.

In our large cohort, non-scheduled surgery, early and late discharge was demonstrated to be risk factors for mortality. The present study adds to previous studies dealing with the risk of hip fracture surgery. Confounding by indication is a major problem in observational studies to describe the effect of interventions. A cluster randomized clinical trial comparing different clinical set ups is warranted.
